# QiSampler: evaluation of scoring schemes for high-throughput datasets using a repetitive sampling strategy on gold standards

**DOI:** 10.1186/1756-0500-4-57

**Published:** 2011-03-09

**Authors:** Jean F Fontaine, Bernhard Suter, Miguel A Andrade-Navarro

**Affiliations:** 1 Max Delbrück Center for Molecular Medicine, Robert-Rössle-Strasse 10, 13125 Berlin, Germany

## Abstract

**Background:**

High-throughput biological experiments can produce a large amount of data showing little overlap with current knowledge. This may be a problem when evaluating alternative scoring mechanisms for such data according to a gold standard dataset because standard statistical tests may not be appropriate.

**Findings:**

To address this problem we have implemented the QiSampler tool that uses a repetitive sampling strategy to evaluate several scoring schemes or experimental parameters for any type of high-throughput data given a gold standard. We provide two example applications of the tool: selection of the best scoring scheme for a high-throughput protein-protein interaction dataset by comparison to a dataset derived from the literature, and evaluation of functional enrichment in a set of tumour-related differentially expressed genes from a thyroid microarray dataset.

**Conclusions:**

QiSampler is implemented as an open source R script and a web server, which can be accessed at http://cbdm.mdc-berlin.de/tools/sampler/.

## Findings

### Background

Due to their large size and complexity, the processing and analysis of data produced by high-throughput molecular technologies requires the application of computer programs and algorithms. For example, the transcriptome of a cell can be assayed with mRNA microarrays, full genomes can be quickly sequenced using deep-sequencing technologies, and data on thousands of protein-protein interactions (PPIs) can be generated using high-throughput yeast two-hybrid screening or mass spectrometry [1,2]. As these technologies produce a huge amount of data, often from samples or conditions never studied before, evaluation of the significance of such results is challenging.

When evaluating the results of a new high-throughput experiment, a biologist's first reaction is often to compare the experimental results to a golden set built from his knowledge and from the literature [3,4]. This golden set would allow the identification of known (previously described in the literature) and novel (not described in the literature) results in the data set, and it would allow the evaluation of different scoring schemes (e.g. experimental parameters, confidence scores or statistical tests) that can be used to prioritize the results. The systematic comparison of prioritizations from different scoring schemes may positively impact the results of a study (e.g. by helping to select appropriate confirmatory experiments) or suggest changes in experimental protocols.

However, it is often the case that high-throughput datasets overlap minimally with available golden sets. For example, in the case of PPI data, a golden set composed of tens of thousands of curated human PPIs can be built from public databases. While this at first sounds like a large golden set, the total interaction space contains hundreds of millions of potentially interacting protein pairs [2,5]. In cases such as this, standard statistical tests may not be appropriate for evaluating a dataset. Therefore, alternative scoring schemes including comparisons of biological attributes such as gene expression or Gene Ontology terms have been used instead [3,6,7]. Yet, the application of these methods to a new dataset requires programming skills and specific statistical knowledge.

We have implemented the QiSampler tool to systematically evaluate several scoring schemes for high-throughput experiments versus given golden sets using a sampling strategy. To demonstrate QiSampler's usefulness, we applied the algorithm to a public PPI dataset to select the experimental score that best prioritizes the data, and to a public microarray dataset to evaluate a functional enrichment in a set of differentially expressed genes.

### Algorithm

The main input to the QiSampler algorithm is a table with at least three pieces of information for each experimental result, or "item": a label describing the item (e.g. gene name, PPI description, etc.), an indication of whether the item is in the golden set ("known") or not ("novel") encoded as '1' and '0' respectively, and one or more scores for the item that was calculated using a scoring scheme that you wish to evaluate. The scores can be integers or decimal numbers, and are expected to correlate positively with the significance of the item. In addition to this table, the user must also provide the number of repetitions N (a positive integer value), and the sampling rate SR ∈ ]0, 1] used to define a sample size S = | number of known items * SR |.

**Figure 1:**
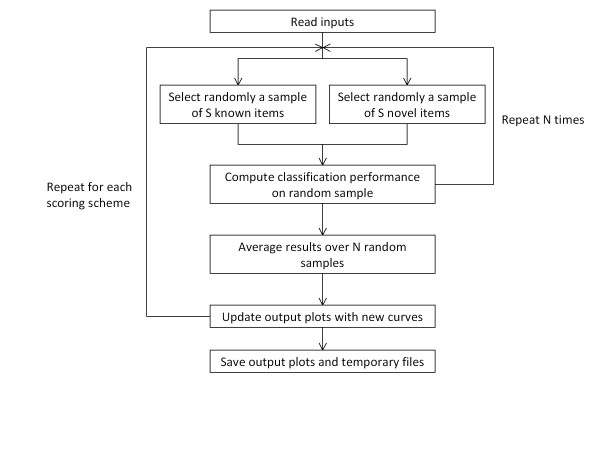
**Flow chart of the QiSampler algorithm**. The data to be processed (all known and novel items and corresponding scoring schemes), and the values of S and N are set from the inputs (see algorithm section for details).

The QiSampler algorithm works by assessing, for each column of alternative scores, whether high values are assigned preferentially to known cases. This is done by comparing the values given to known items to randomly chosen novel items. The algorithm can be described as follows (see also Figure 1):

1. select a random sample of size S from the known items in the dataset

2. select a random sample of the same size S from the novel items in the dataset

3. compute the classification performance on the random sample for a given scoring scheme

4. repeat steps 1 to 3 N times

5. compute the average classification performance over the N random samples

6. update output plots with averaged performance curves over the N repetitions

7. repeat steps 1 to 6 for each scoring scheme

**Figure 2:**
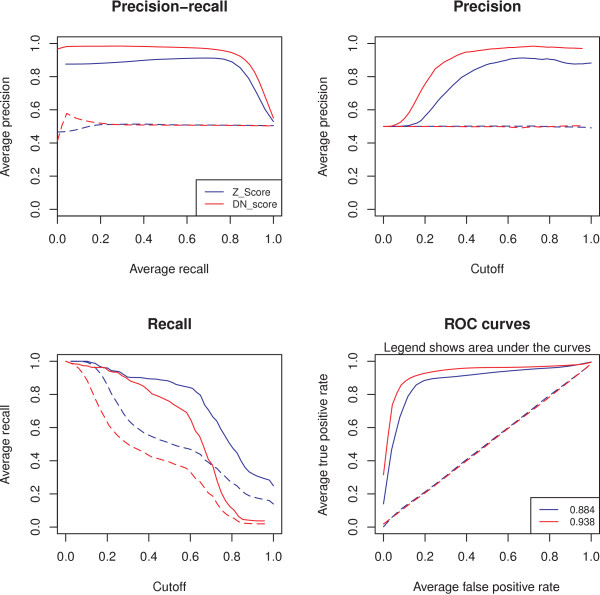
**Scores comparison**. These graphs produced by QiSampler show the average performance of two scores (scaled to [0,1]), used to select PPIs from the same experimental dataset [10]. Performance was averaged over 1000 repetitions with a sampling rate of 25%. Dashed lines represent randomized data. Based on the Precision-recall and ROC graphs, the normalized DN score performs better than the z-score and a cut-off close to 0.3 would produce optimal values of recall and precision.

Performances are summarized using four graphical plots: precision-recall, precision-cutoff, recall-cutoff, and receiver operating characteristic (ROC) curves (See Figure 2). Classification statistics are defined as follows: given a score cutoff for item selection, true positive items (TP) are defined as known items that are selected, false positive items (FP) as novel items that are selected, true negative items (TN) as novel items that are not selected, and false negative items (FN) as known items that are not selected. Then, the following classification performance measures are used: recall = TP/(TP + FN), true positive rate = recall, false positive rate = FP/(FP + TN), and precision = TP/(TP + FP) [8,9]. Examination of the graphs permits comparison of the scoring systems, and could suggest optimal cutoff values for specific applications.

When running QiSampler, the user can test the effect of changing the SR parameter, which defines the sample size S. This is useful to detect whether the results of the algorithm could be biased by the high scores of a few true positives. The observation of a drastic decrease in performance when changing SR from high to low (e.g. from 90% to 10%) would tell that just a few true positive items have higher scores than the random selection. On the contrary, stable performance when varying SR would tell that most of the true positive items have comparable scores. Another parameter that is defined by the user is the number N of repetitions of the test. The procedure should be repeated a minimal number of times to produce a good estimate of the performance (we would suggest at least 100 times). Lower values may have to be used if the computational requirements are too high.

### Example of application to a PPI dataset

We have downloaded data from a proteomics study where interaction partners of 75 deubiquitinating enzymes were defined using protein purification, immunoprecipitation and tandem mass spectrometry [10]. The full dataset consisted of 26,803 protein pairs evaluated for potential interaction using two different scores: the z-score and the DN confidence score, the latter introduced by the authors to score interactions using information from parallel nonreciprocal datasets. The superiority of the DN score was shown only for a few examples [10].

Here, we have used QiSampler to perform a systematic statistical evaluation of the two scoring systems in this dataset (Figure 2). The dataset included 105 known pairs that have been previously described in the literature (as defined in [10]). We selected N = 1000 repetitions and SR = 25% (equivalent to 26 pairs). The running time was approximately 40 minutes, but it can vary drastically from a few seconds to a few hours when different parameters are used (Table 1).

**Table 1:** Average running times on the full dataset

Sampling rate	0.25	0.75	1
Running time for 10 repetitions	00:00:04	00:00:07	00:00:11
Running time for 100 repetitions	00:00:54	00:03:30	00:04:46
Running time for 1000 repetitions	00:40:15	03:09:14	05:09:52

The full dataset contained 26,803 protein pairs including 105 known in the literature. Times were averaged over two runs and were recorded on an AMD Opteron (64 bits, 2.3 GHz) processor-based computer.

For comparison, the DN score was log transformed (logarithm base 10) and both scores (z-score and DN score) were then scaled to [0,1]. The scaled version S_scaled _of a score S was defined as S_scaled _= (S - S_min_)/( S_max _- S_min_), where S_max _and S_min _are the maximal and minimal value of S respectively. The DN score (blue curves) showed higher precision but lower recall than the z-score (red curves). Nevertheless, the balance between precision and recall (precision-recall curve), or true positive rate and false positive rate (ROC curve) was better for the DN score, showing its superiority. Both scores were better than random controls in the four plots. Results were stable when varying the sampling rate from 10% to 100% (data not shown).

### Example of application to a microarray dataset

We downloaded from the Gene Expression Omnibus database [11] a microarray dataset (identifier: GSE6339) containing normalized gene expression values of human thyroid samples, and extracted data from 30 oncocytic thyroid adenoma (OTA) and 24 wild type (WT) samples [12]. OTA cells are characterized by an accumulation of mitochondria [13]. We used QiSampler to see if genes disregulated in OTA samples were significantly related to oxidoreductase activity, which is related to mitochondrial function [14].

**Figure 3:**
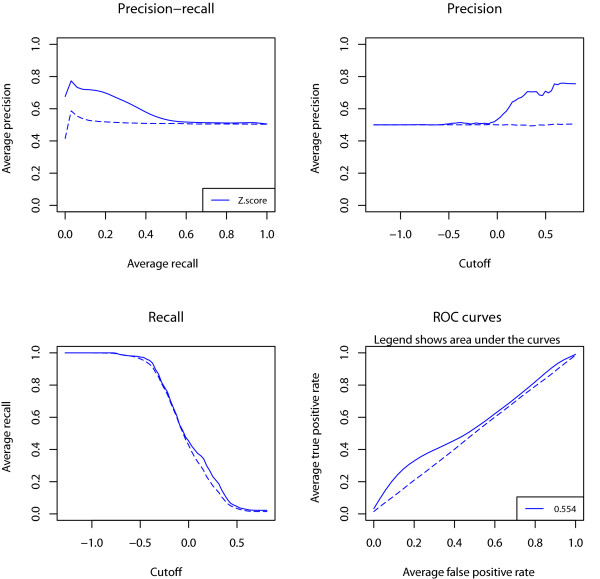
**Functional enrichment in differentially expressed genes**. These graphs produced by QiSampler show the average performance of the z-score comparing gene expression values between 30 OTA and 24 WT samples to select gene probes related to oxidoreductase activity in a thyroid microarray gene expression dataset [12]. The performance was averaged over 1000 repetitions with a sampling rate of 25% representing 34/137 known items and 34/3684 novel items. Dashed lines represent randomized data. The precision increases with the z-score cutoff indicating functional enrichment in the upregulated genes.

Z-scores comparing expression values between OTA and WT samples were computed for 3,821 gene probes with associated Gene Ontology (GO) annotations and a number of missing values less than or equal to 27 (representing 50% of the samples). There were 137 (3.6%) gene probes associated to "oxidoreductase activity" annotation (GO:0016491). We selected N = 1000 repetitions and SR = 25% (equivalent to 34 gene probes) to produce classification performance plots by QiSampler (Figure 3).

The separation of the precision-recall curve from the random curve shows that genes related to oxidoreductase activity tend to be upregulated demonstrating the initial hypothesis. The shape of the ROC curve is probably due to the fact that not all the genes related to oxidoreductase activity are involved in OTA and that the set of upregulated genes is expected to include genes related to other cellular and molecular processes such as apoptosis or mitochondrial homoeostasis [13,15].

### Implementation

The R script is designed for R 2.9.0 [16] and plots are generated by the ROCR package [9]. The script is used in command-line mode. To facilitate access to the algorithm we also implemented the algorithm as a public web tool, programmed using JavaScript, HTML 4 and Perl 5.8.8, but it operates with some query-size limits due to restricted computing power. Web pages were tested on Linux or Windows using Firefox 3.6.8, Google Chrome 5.0, and Internet Explorer 8.0.

## Discussion

QiSampler can systematically evaluate the classification performance of experimental scores in comparison to gold standards and to random controls. A given scoring scheme can be identified as relevant if it produces better classification performance than randomly generated scores. If the user provides multiple scoring schemes, their performance can be easily compared, as illustrated in Figure 2.

To illustrate QiSampler's usefulness we applied it to the analysis of a high-throughput PPI dataset (26,803 protein pairs) produced by mass spectrometry experiments that has little overlap with the PPI literature (105 known interacting pairs) [10]. In the original publication, a particular score was designed for mass spectrometry data (DN score), which accounts for protein abundance and performs better than the z-score in the selection of protein spectrometry results [10]. Accordingly, the QiSampler was able to reproduce the superiority of the DN score over the z-score (Figure 2). In a second application, we used QiSampler to demonstrate the enrichment in functions related to oxidoreductase activity in genes upregulated in oncocytic thyroid adenomas from a microarray dataset (Figure 3).

Although QiSampler was created to process datasets with little overlap to the literature, it will not be able to process a dataset with very few known cases or with too few different score levels (e.g. a binary score) due to a limitation in a function of the ROCR package. We recommend running the procedure with datasets having at least 10 known cases, the more the better, and scored with continuously distributed values.

The algorithm may have long run times when processing large datasets (Table 1), and due to restricted computing power the web server operates with some restrictions on the size of the query. To avoid this problem, an open-source R script is provided which allows one to use QiSampler locally as a command line program without limited inputs.

Plans for the future include an improved web server able to process large datasets. Automatic computation of optimal score cutoffs could also be useful, though different applications may require different cutoffs, e.g. giving priority to higher recall accepting poor precision (e.g. genetic disease screenings) or to higher precision accepting poor recall (e.g. identification of disease markers). Result reliability when varying the sampling rate could be automatically computed, though running QiSampler twice is sufficient to see such an effect, for example comparing sampling rates of 10% and 90%. Finally, to further simplify the use of the QiSampler tool, which requires the user to provide the scores and the identification of the known items, we will implement optional pre-computed score systems and golden standards upon request from users if these are commonly used in the research community.

In conclusion, QiSampler can be used for the selection of the most useful experimental scores or parameters. Simplicity of the input format allows the use of QiSampler with various dataset types, such as PPI, gene-expression microarray, or deep sequencing datasets.

## Availability and requirements

Project name: QiSampler

Project home page: http://cbdm.mdc-berlin.de/tools/sampler/

Operating system(s): platform independent

Programming language: R, Perl, HTML, and JavaScript

Other requirements: either a modern web browser or R 2.9.0 and the ROCR package

License: BSD license

Any restrictions to use by non-academics: none

## List of abbreviations

PPI: protein-protein interaction.

## Competing interests

The authors declare that they have no competing interests.

## Authors' contributions

JFF conceived of the study, designed the study, carried out implementation and statistical analysis, and drafted the manuscript. BS participated in the statistical analysis. MAAN participated in the study design and collaborated in the writing of the manuscript. All authors read and approved the final manuscript.
